# Association of D-dimer and acute kidney injury associated with rhabdomyolysis in patients with exertional heatstroke: an over 10-year intensive care survey

**DOI:** 10.1080/0886022X.2021.2008975

**Published:** 2021-11-28

**Authors:** Conglin Wang, Baojun Yu, Ronglin Chen, Lei Su, Ming Wu, Zhifeng Liu

**Affiliations:** a Department of Critical Care Medicine, General Hospital of Southern Theatre Command of Peoples Liberation Army, Guangzhou, China; b The First School of Clinical Medicine, Southern Medical University, Guangzhou, China; c Department of Critical Care Medicine, Bao’an People's Hospital, Shenzhen, China; d Department of Critical Care Medicine, Central People’s Hospital of Longgang, Shenzhen, China; e Key Laboratory of Hot Zone Trauma Care and Tissue Repair of Peoples Liberation Army, General Hospital of Southern Theatre Command of Peoples Liberation Army, Guangzhou, China; f Department of Critical Care Medicine and Hospital Infection Prevention and Control, The Second People’s Hospital of Shenzhen & First Affiliated Hospital of Shenzhen University, Health Science Center, Shenzhen, China

**Keywords:** D-dimer, acute kidney injury, rhabdomyolysis, exertional heatstroke

## Abstract

Patients with rhabdomyolysis (RM) following exertional heatstroke (EHS) are often accompanied by dysfunction of coagulation and acute kidney injury (AKI). The purpose of this study was to investigate the relationship between D-dimer and AKI in patients with RM following EHS. A retrospective study was performed on patients with EHS admitted to the intensive care unit over 10-year. Data including baseline clinical information at admission, vital organ dysfunction, and 90-day mortality were collected. A total of 84 patients were finally included, of whom 41 (48.8%) had AKI. AKI patients had more severe organ injury and higher 90-day mortality (34.1 *vs.*0.0%, *p* < 0.001) than non-AKI patients. Multivariate logistic analysis showed that D-dimer (OR 1.3, 95% CI 1.1–1.7, *p* = 0.018) was an independent risk factor for AKI with RM following EHS. Curve fitting showed a curve relationship between D-dimer and AKI. Two-piecewise linear regression showed that D-dimer was associated with AKI in all populations (OR 1.3, 95% CI 1.2–1.5, *p* < 0.001) when D-dimer <10.0 mg/L, in RM group (OR 1.3, 95% CI 1.1–1.5, *p* < 0.001) when D-dimer >0.4 mg/L, in the non-RM group (OR 6.4, 95% CI 1.7–23.9, *p* = 0.005) when D-dimer <1.3 mg/L and D-dimer did not increase the incidence of AKI in the non-RM group when D-dimer >1.3 mg/L. AKI is a life-threatening complication of RM following EHS. D-dimer is associated with AKI in critically ill patients with EHS. The relationship between D-dimer and AKI depends on whether RM is present or not.

## Introduction

An unusual heatwave is raging in the northwest of the United States and the western of Canada. According to incomplete statistics, up to 30 June 2021, more than 500 people were killed by heatstroke for high temperatures. Heatstroke has become an important public health event, which can be classified as exertional heat stroke (EHS) and classic heatstroke (CHS) [1]. EHS, defined as an internal body temperature >40 °C with associated brain impairment, is directly related to strenuous physical activity and is considered a life-threatening medical emergency, requiring prompt recognition, management, and care to ensure survival [1]. EHS is often complicated by rhabdomyolysis (RM), which leads to multiple organ dysfunctions including acute kidney injury (AKI) [2]. It has been reported that RM occurred in ∼26 000 patients in the United States each year, of whom 7–10% of RM patients may develop into AKI [3]. Research showed that Sequential Organ Failure Assessment (SOFA) score was an independent risk factor affecting the survival of patients, based on reducing the SOFA score may be pivotal for reducing the mortality of EHS complicated with AKI [4,5]. The study showed that the incidence of disseminated intravascular coagulation (DIC) reduced by severe heatstroke was 34.5%, but the incidence increased to 51.6% when combined with AKI [5].

In animal research, due to the release of damage-associated molecular patterns (DAMPs) such as high mobility group box-1 protein (HMGB1), EHS often accompanied by coagulation dysfunction, just similar to sepsis-associated coagulopathy [6]. What’s more, a clinical case-control study found that D-dimer levels were higher in patients with EHS complicated with DIC than those without DIC [7]. It has also been reported that D-dimer as a coagulation-related marker could be used as an effective factor for predicting the occurrence of AKI [8,9]. However, there have been no clinical studies on whether D-dimer can be a predictor of AKI in EHS patients with RM. Therefore, a retrospective observational study was designed in a tertiary-care teaching hospital in southern China over a 10-year period. The aim of the study is to investigate the relationship between D-dimer and AKI, which guides anticoagulation based on D-dimer level and prevents the occurrence of AKI in the future.

## Methods

### Study design and participants

The single-center retrospective observational study was designed in the intensive care unit (ICU) of the General Hospital of Southern Theater Command of Peoples Liberation Army from January 2008 to June 2019. The inclusion criterion was referred to in previous articles published by our team as follows [5]: patients with ‘exertional heatstroke’ caused by strenuous exercise under a high-temperature and high-humidity environment. The diagnostic criteria of exertional heatstroke were as follows [1]: a history of strenuous exercise while exposure to high temperature, high humidity; a clinical syndrome causing hyperthermia (central temperature over 40 °C); nervous system dysfunction (including coma, cognitive impairment, delirium, etc.); or systemic organ dysfunction. The exclusion criteria were as follows: (1) absence of key indicators data, (2) death or discharge within 24 h after admission, (3) a previous history of organ dysfunction, such as chronic kidney disease, and (4) incomplete outcome evaluation data obtained through telephone follow-up.

All patients were received comprehensive treatment according to their condition, such as body cooling, administering fluids, anti-inflammation at the same time, and organ function supports were performed for patients with RM as well as AKI if necessary under clinical guidelines, including appropriate hydration, alkalization of urine, continuous renal replacement therapy (CRRT) and so on [5].

### Research procedures

The basic characteristics of the patients were recorded, including the Acute Physiology and Chronic Health Evaluation II (APACHE II) score, SOFA score, Glasgow Coma Scale (GCS) score, ages, and inflammatory and organ function indicators at admission. The indicators included blood count [white blood cell (WBC), neutrophil, lymphocyte, monocytes, platelets, mean platelet volume, platelet distribution width], liver function markers [total bilirubin (TBIL), alanine aminotransferase (ALT), aspartate aminotransferase (AST)], kidney function markers [blood urea nitrogen (BUN), serum creatinine (Scr), Cystatin C], cardiac markers [creatine kinase (CK), MB isoenzyme of creatine kinase (CK-MB), myoglobin (MB), cardiac troponin I (cTNI)], clotting factors [prothrombin time (PT), international normalized ratio (INR), activated partial thromboplastin time (APTT), thrombin time (TT), fibrinogen (FIB), D-dimer], C-reactive protein (CRP), and procalcitonin (PCT). All the patients were assigned to the AKI group or the non-AKI group according to the presence of AKI. Survival time was defined as the duration from onset to death; when the survival time was longer than 90 days, it was recorded as 90 days. The main results, including 90-day mortality, ICU time, survival time, and the total cost during hospitalization, were analyzed. This study was approved by the Research Ethics Committee of the General Hospital of Southern Theater Command of Peoples Liberation Army (HE-2020-09). Considering the retrospective study design and depersonalization of the data, the Ethics Committee agreed to waive the requirement for written informed consent but required that the patients be informed of the study details during the telephone follow-up.

### Definitions

1. RM [10]: This study adopted the current consensus opinion that CK > 1000 U/L was considered elevated CK, while an increase in CK due to cardiogenic shock (CK-MB/CK < 5%) was excluded. Clinical manifestations included general fatigue, muscle soreness, and soy sauce-like urine.
2. AKI [11]: KDIGO standard: Scr increase to ≥26.5 μmol/L (≥0.3 mg/dl) within 48 h, Scr increase to ≥1.5 times the baseline within 7 days, or urine output <0.5 ml/(kg·h) for 6 h.
3. Lymphocytopenia [12]: absolute lymphocytes <0.8 × 10^9^/L.
4. DIC [13]: International Society for Thrombosis and Haemostasis (ISTH) standard: An ISTH score ≥5 points.
5. Acute hepatic injury (AHI) [14]: Plasma TBIL ≥34.2 μmol/L and INR ≥1.5, or with any grade of hepatic encephalopathy.

### Statistical analysis

The continuous variables conforming to a normal distribution are expressed as $\bar{x}\pm s.$ For continuous variables that did not conform to a normal distribution, count data and ordinal data are represented as median and interquartile ranges (IQRs). Count data were compared using multiple independent samples nonparametric Kruskal–Wallis *H* tests, and measurement data for intergroup comparisons were analyzed by nonparametric Mann–Whitney *U* tests. A univariate analysis was to analyze significant indicators. Indicators with a *p*-value <0.1 were included in the multivariate logistic regression (LR) model, and forward stepwise regression was used to gradually eliminate each variable. Using a two-piecewise linear regression model to curve fitting. Statistical analyses were performed by SPSS Windows version 23.0 (SPSS Inc., Chicago, IL, USA), Empower (R) (http://www.empowerstats.com, X&Y solutions, Inc., Boston, MA, USA). A two-tailed *p*-value less than 0.05 was considered statistically significant.

## Results

### Baseline of patients with RM induced by EHS

**Figure 1.**
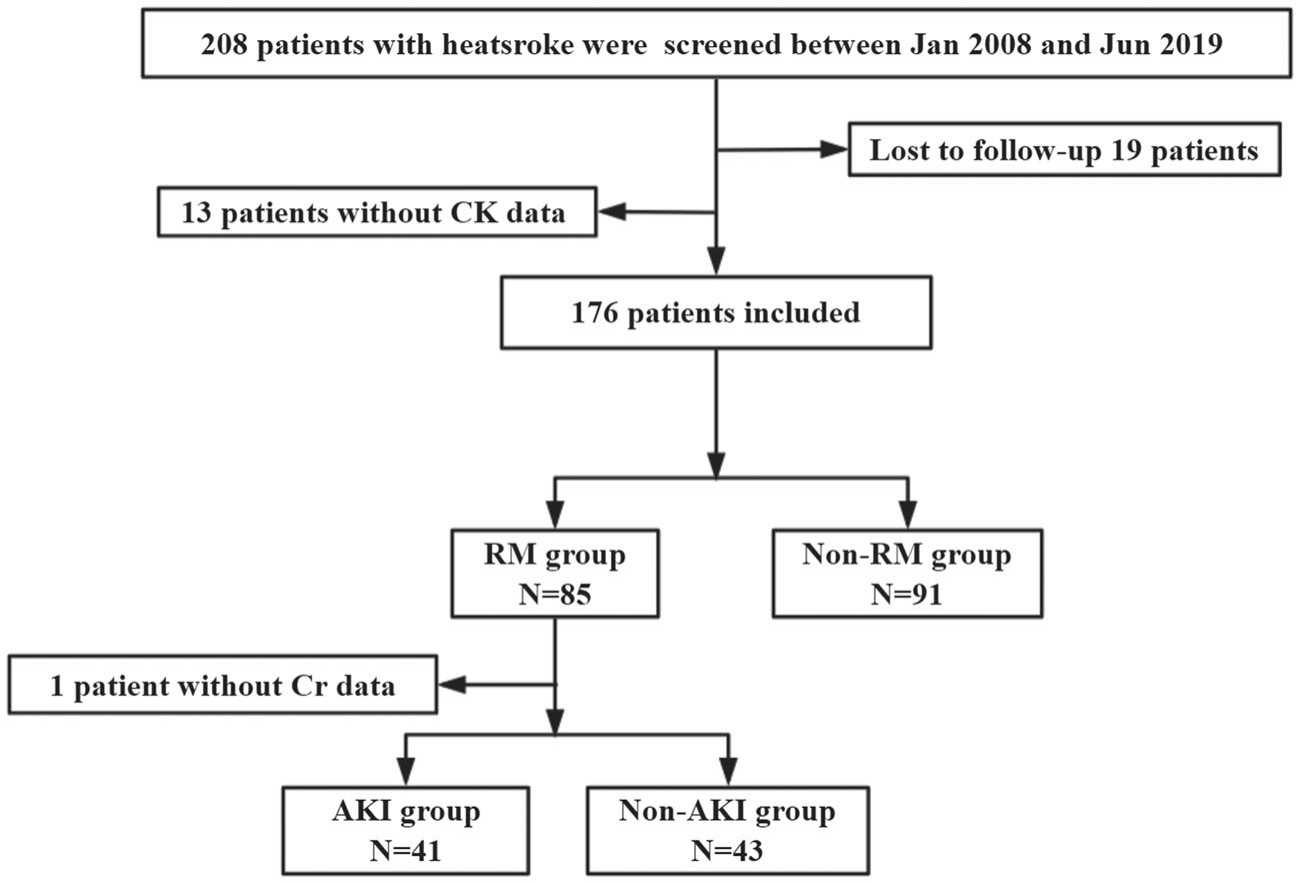
Flow chart of all excluded and included patients.

A total of 208 patients were screened, of whom 32 patients were excluded because they were lost to follow-up or with missing clinical data. Finally, 176 patients were included and 85 patients who were complicated with RM. Because 1 patient had a missing Scr date, 84 patients were included finally. Of these, 41 patients (48.8%) had AKI and 43 patients (51.2%) did not have AKI (Figure 1). There was no statistically significant difference in age between AKI and non-AKI patients (23.0 *vs.* 21.0, *p* = 0.056). Compared with the patients without AKI, the patients with AKI had significantly higher APACHE II (17.0 *vs.* 9.0, *p* < 0.001) and SOFA scores (9.0 *vs.* 3.0, *p* < 0.001); lower GCS scores (8.0 *vs.* 12.0, *p* = 0.006) and platelets count [80.0 *vs.* 152.0 (10^9^/L), *p* < 0.001]; prolonged PT [23.0 *vs.* 16.3 (s), *p* = 0.017] and TT [21.8 *vs.* 17.5 (s), *p* = 0.045]; FIB [2.1 *vs.* 2.6 (g/L), *p =* 0.002] was decreased, while INR (2.0 *vs.* 1.5, *p* < 0.001) and D-dimer [10.0 *vs.* 1.1 (mg/L), *p* < 0.001] were increased. However, there were no significant differences in the incidence of lymphocytopenia (52.5 *vs.* 37.2%, *p =* 0.161) or AHI (74.4 *vs.* 75.6%, *p =* 0.897). In addition, patients with AKI had a significantly higher incidence of DIC (75.0 *vs.* 31.2%, *p* < 0.001), longer ICU time [7.5 *vs.* 5.0 (d), *p =* 0.023], 90-day mortality was significantly increased (34.1 *vs.* 0.0%, *p* < 0.001), and the total cost of hospitalization was significantly higher [113 013.1 *vs.* 36 748.7 (Renminbi, RMB), *p* < 0.001] (Table 1).

**Table 1.** Comparisons of clinical characteristics between non-AKI and AKI patients with RM induced by EHS.

	Non-AKI (*n* = 43)	AKI (*n* = 41)	*p*-Value
APACHE II score, median (IQR)	9.0 (7.0–14.0)	17.0 (11.0–22.0)	<0.001
SOFA score, median (IQR)	3.0 (2.0–4.0)	9.0 (5.0–11.0)	<0.001
GCS score, median (IQR)	12.0 (9.0–14.0)	8.0 (6.0–12.0)	0.006
Age (years), median (IQR)	21.0 (19.0–26.5)	23.0 (20.0–28.0)	0.056
WBC (1 × 10^9^/L), median (IQR)	11.6 (8.9–14.3)	12.0 (9.2–14.9)	0.209
Neutrophil (1 × 10^9^/L), median (IQR)	8.8 (7.0–12.5)	10.0 (6.9–13.3)	0.187
Lymphocyte (1 × 10^9^/L), median (IQR)	1.1 (0.7–1.7)	0.7 (0.4–1.7)	0.759
Monocytes (1 × 10^9^/L), median (IQR)	0.7 (0.5–1.0)	0.7 (0.4–1.0)	0.549
Platelets (1 × 10^9^/L), median (IQR)	152.0 (89.0–199.0)	80.0 (38.0–115.5)	<0.001
Mean platelet volume, median (IQR)	10.7 (10.0–11.5)	10.8 (10.1–11.3)	0.987
Platelet distribution width, median (IQR)	12.6 (11.1–13.6)	12.5 (11.1–14.3)	0.514
TBIL (µmol/L), median (IQR)	16.6 (12.4–25.8)	24.4 (14.2–62.9)	0.079
ALT (U/L), median (IQR)	54.0 (24.5–333.0)	148.0 (57.0–1771.0)	0.014
AST (U/L), median (IQR)	118.0 (64.0–483.0)	290.0 (115.0–1587.0)	0.014
BUN (mmol/L), median (IQR)	5.1 (4.2–6.1)	7.8 (6.4–10.3)	<0.001
Scr (µmol/L), median (IQR)	93.0 (78.0–107.5)	187.0 (151.0–263.0)	<0.001
Cystatin C (mg/L), median (IQR)	0.9 (0.8–1.0)	1.2 (1.0–1.8)	<0.001
CK (U/L), median (IQR)	2434.0 (1462.5–4714.5)	3506.0 (1614.0–7894.0)	0.780
CK-MB (ng/ml), median (IQR)	63.0 (41.5–107.0)	89.0 (56.5–209.5)	0.040
MB (ng/ml), median (IQR)	466.0 (174.0–1000.0)	1000.0 (979.8–1000.0)	<0.001
cTNI (pg/ml), median (IQR)	60.0 (11.0–210.0)	580.0 (195. 0–1103.5)	0.018
PT (s), median (IQR)	16.3 (15.3–18.9)	23.0 (17.1–36.9)	0.017
INR (median (IQR)	1.5 (0.6) 1.3 (1.2–1.6)	2.0 (1.4–3.7)	<0.001
APTT (s), median (IQR)	41.0 (36.5–46.8)	49.9 (38.3–85.4)	0.299
TT(s), median (IQR)	17.5 (16.6–23.4)	21.8 (17.1–36.5)	0.045
FIB (g/L), median (IQR)	2.6 (2.3–3.0)	2.1 (1.4–2.6)	0.002
D-dimer (mg/L), median (IQR)	1.1 (0.6–3.9)	10.0 (3.7–14.4)	<0.001
CRP (mg/dl), median (IQR)	4.2 (3.2–7.7)	3.4 (2.4–6.4)	0.366
PCT (ng/ml), median (IQR)	2.5 (1.1–4.3)	3.7 (1.6–6.8)	0.734
MB ≥ 1000 ng/ml, *N* (%)	10/37 (27.0%)	26/35 (74.3%)	<0.001
Lymphocytopenia, *N* (%)	16/43 (37.2%)	21/40 (52.5%)	0.161
DIC, *N* (%)	10/32(31.2%)	24/32(75.0%)	<0.001
AHI, *N* (%)	31/41 (75.6%)	29/39 (74.4%)	0.897
90-day mortality, *N* (%)	0/43 (0.0%)	14/41 (34.1%)	<0.001
ICU time (d), median (IQR)	5.0 (3.0–7.5)	7.5 (5.0–13.5)	0.023
Survival time (d), median (IQR)	90.0 (90.0–90.0)	90.0 (9.0–90.0)	<0.001
Hospitalization costs (RMB), median (IQR)	36 748.7 (22 408.4–64 406.8)	113 013.1 (45 869.3–224 580.8)	<0.001

APACHE II: Acute Physiology and Chronic Health Evaluation II; SOFA: Sequential Organ Failure Assessment; GCS: Glasgow Coma Scale; WBC: white blood cell; TBIL: total bilirubin; ALT: alanine aminotransferase; AST: aspartate aminotransferase; BUN: blood urea nitrogen; Scr: serum creatinine; CK: creatine kinase; CK-MB: MB isoenzyme of creatine kinase; MB: myoglobin; cTNI: cardiac troponin I; PT: prothrombin time; INR: international normalized ratio; APTT: activated partial thromboplastin time; TT: thrombin time; FIB: fibrinogen; CRP: C-reactive protein; PCT: procalcitonin. DIC: disseminated intravascular coagulation; AHI: acute hepatic injury.

### Risk factors for AKI and the relationship between D-dimer and AKI in patients with EHS

**Figure 2.**
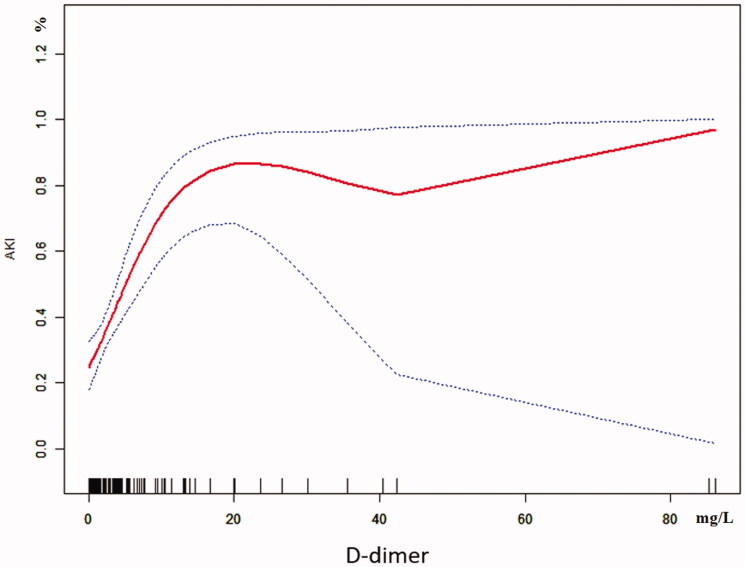
Curve fitting of D-dimer in predicting AKI in EHS.

**Figure 3.**
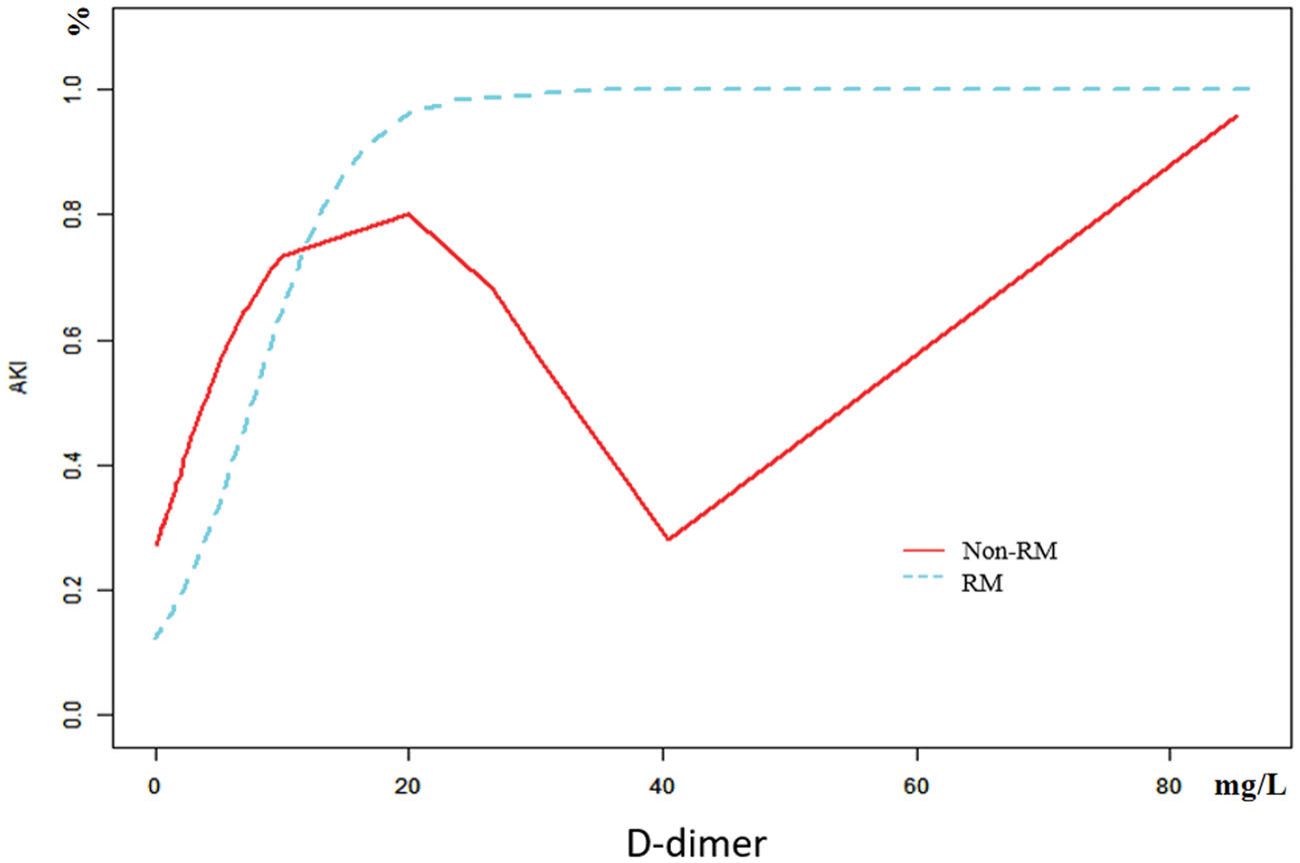
Curve fitting of D-dimer in predicting AKI between RM and non-RM in EHS.

The univariate analysis showed that D-dimer and GCS were closely related to AKI in RM patients following EHS, and the differences were statistically significant (both *p* < 0.05). What’s more, multivariate logistic regression showed that the D-dimer (OR 1.3, 95% CI 1.1–1.7, *p* = 0.018) was the independent risk factor for AKI in RM patients induced by EHS (Table 2). Curve fitting showed a curve relationship between D-dimer and AKI complicated with EHS (Figure 2). According to the two-piecewise linear regression model, the risk of AKI increased by 30% with each increase of 1 mg/L when D-dimer was less than 10.0 mg/L (OR 1.3, 95% CI 1.2–1.5, *p* < 0.001), and saturation effect was observed when D-dimer was greater than 10.0 mg/L in the whole patients. Moreover, in patients of EHS complicated with RM, D-dimer less than 0.4 mg/L has no significant effect on the incidence of AKI. But the risk of AKI increases by 30% with each increase of 1 mg/L when D-dimer is greater than 0.4 mg/L (OR 1.3, 95% CI 1.1–1.5, *p* < 0.001), and the saturation effect is no longer present. In patients of EHS without RM, the risk of AKI increased by 5.4 times for each increase of 1 mg/L when D-dimer was less than 1.3 mg/L (OR 6.4, 95% CI 1.7–23.9, *p* = 0.005). And when D-dimer was greater than 1.3 mg/L, the continuous increase of D-dimer did not increase the incidence of AKI (Table 3, Figure 3).

**Table 2.** Risk factors for AKI with RM induced by EHS.

	Univariate OR (95%CI) *p-*value	Multivariate OR (95%CI) *p-*value
GCS	0.8 (0.7, 0.9) 0.009	1.0 (0.7, 1.4) 0.972
PT	1.0 (1.0, 1.1) 0.044	1.0 (0.9, 1.2) 0.671
MB	1.0 (1.0, 1.0) 0.003	1.0 (1.0, 1.0) 0.518
D-dimer	1.3 (1.1, 1.5) <0.001	1.3 (1.1, 1.7) 0.018
Platelets	1.0 (1.0, 1.0) 0.002	1.0 (1.0, 1.0) 0.837

GCS: Glasgow Coma Scale; PT: prothrombin time; MB: myoglobin.

**Table 3.** D-dimer in predicting AKI with two-piecewise linear regression model in non-RM and RM patients.

	Total	Non-RM	RM
	OR (95%CI) *p-*value	OR (95%CI) *p-*value	OR (95%CI) *p-*value
Model I			
One linear regression coefficient	1.1 (1.1, 1.2) <0.001	1.1 (1.0, 1.1) 0.114	1.3 (1.1, 1.5) <0.001
Model II			
K	10	1.3	0.4
<K regression coefficient 1	1.3 (1.2, 1.5) <0.001	6.4 (1.7, 23.9) 0.005	0.0 (0.0, 50.7) 0.285
>K regression coefficient 2	1.0 (1.0, 1.1) 0.849	1.0 (1.0, 1.1) 0.591	1.3 (1.1, 1.5) <0.001
Difference between the regression coefficient 2 and 1	0.8 (0.7, 0.9) <0.001	0.2 (0.0, 0.6) 0.006	147.3 (0.0, 872159.1) 0.26
Predicted value of Y at K	1.5 (0.6, 2.4)	0.3 (−0.5, 1.1)	−1.5 (−2.5, −0.6)
Logarithmic LR test	0.002	0.004	0.267

K: Kurtosis; LR: logistic regression.

## Discussion

EHS is the most severe exertional heat illness. It may predispose to be complicated with RM, aggravating multiple organ dysfunction such as AKI, DIC, and myocardial injury [5,7,15]. This study found that in patients with RM induced by EHS, 48.8% of them developed AKI. Compared with non-AKI patients, AKI patients had higher APACHE II and SOFA scores, lower GCS scores, and a higher incidence of DIC (all *p* < 0.05). At the cellular level, the physiological response to thermal stress involves various cytokines, including tumor necrosis factor α (TNF-α), interleukin-1 (IL-1), and interleukin-6 (IL-6), as well as heat shock proteins (HSPs). Endothelial cells mediate the inflammatory response, activate platelets, release tissue factors to aggravate coagulation disorders [16]. It has also shown that complement was involved in the interaction with coagulation [17]. As a common complication in EHS, DIC can contribute to long-term tissue damage leading to multiple organ failures including AKI. If suitable treatment is not provided in time, it will often lead to death [18–21].

The laboratory diagnosis of ISTH about DIC includes platelets, D-dimer, fibrin degradation products, FIB, and PT [13]. It has also been reported that D-dimer as a coagulation-related marker could be used as an effective factor for predicting the occurrence of AKI [8,9,22]. In recent years, it was suggested that the D-dimer concentration is likely to rise in the setting of systemic inflammation and infection [23,24]. In patients with EHS, the relationship between D-dimer and AKI is still unclear, which affects the prevention and treatment of AKI to a certain extent. We found that the D-dimer was the independent risk factor by using multivariate logistic regression. In addition, curve fitting showed a curve relationship between D-dimer and AKI complicated with EHS. To further clarify the relationship between D-dimer and AKI, we adopted the two-piecewise linear regression method to analyze, which showed that D-dimer was associated with AKI in all included patients (OR 1.3, 95% CI 1.2–1.5, *p* < 0.001) when D-dimer <10.0 mg/L. However, elevated D-dimer no longer increases the occurrence of AKI when D-dimer >10.0 mg/L, which showed that D-dimer has a saturation effect on AKI. Based on the fact that RM releases a large number of intracellular substances (including HMGB1, etc.) and MB, which affect the coagulation system and organ function, we used a stratified analysis to analyze the effect of RM on D-dimer and AKI. The results showed that in the RM group when D-dimer >0.4 mg/L (OR 1.3, 95% CI 1.1–1.5, *p* < 0.001) and in the non-RM group when D-dimer < 1.3 mg/L (OR 6.4, 95% CI 1.7–23.9, *p* = 0.005), the incidence of AKI was increased. But there was no augment in the non-RM group when D-dimer >1.3 mg/L (OR 1.0, 95% CI 1.0–1.1, *p* = 0.591). In a word, different D-dimer inflection points could indicate the incidence of AKI to different degrees in different groups. The most significant is that in patients with EHS without RM, the risk of AKI increased by 5.4 times for each 1 mg/L increase in D-dimer <1.3 mg/L.

A significant increase in D-dimer means that coagulation is extensively activated. Microthrombosis leads to energy metabolism disorders in important organs. The significance of anticoagulation therapy is to reduce the D-dimer and excessive consumption of coagulation substances by using heparin, thereby improving the pathological process of the occurrence and development of DIC. Expert consensus on the diagnosis and treatment of heat stroke in China [25] recommends low-dose heparin (1–8 U/kg/h) maintenance based on the patient’s coagulation function and organ function status and adjusts the dose according to the APTT or R time of thrombelastography (TEG) or the activation hemoglutination time (ACT) of coagulation and platelet function analyzer (Sonoclot analyzer) compared with the previous basic value. The experience of the author's team is to use the Sonoclot analyzer at the bedside, combine the blood routine examination and five items of coagulation, and then start the anticoagulant therapy at the same time and dynamically adjust the heparin under the premise of the goal-oriented alternative therapy. If there is active bleeding, heparin should be stopped immediately, and the protamine should be properly used to be neutralized. After the bleeding is basically controlled, the timing of anticoagulation treatment should be evaluated again. D-dimer was associated with AKI with a different cutoff point in different groups. Therefore, in addition to guiding anticoagulation through the coagulation monitoring mentioned above, the use of D-dimer as the target of anticoagulation is a clinical issue worthy of further exploration in EHS complicated with AKI in the future.

A previous study had shown that MB ≥1000 ng/ml is an independent risk factor of AKI. But in patients with EHS complicated with RM, multivariate logistic regression showed there was no difference on MB (OR = 1.0) [5]. Other influential factors should take into consideration. The factors leading to kidney damage induced by EHS mainly include prerenal dehydration [26], blockage of postrenal myoglobin [27], renal vessels (microthrombosis) [28], renal interstitium (inflammation) [29], and some undetermined relevant factors. Moreover, neutrophil extracellular traps [30,31] and platelet activation [32] also play a key role in RM-induced AKI. What’s more, complement activation also plays an important role in driving to AKI in RM, which has shown that complement inhibition represents promising therapeutic strategies [33].

This study has some limitations. It was a single-center retrospective observational study. Since the time of this study is over 10 years, the detection methods of the indicators may be different, resulting in different clinical cutoff points of D-dimer. Because all patients were male and the average age was relatively young, it could not fully reflect the overall conditions of the heatstroke population. Future studies should include multicenter cohorts to expand the sample size to achieve higher-level clinical outcomes.

## Conclusions

RM complicated with AKI in EHS patients had a worse clinical condition and higher 90-day mortality than those without AKI. D-dimer was an independent risk factor for AKI in patients with RM induced by EHS. It is worth further exploration to use D-dimer as the guide anticoagulant treatment target for preventing the occurrence of AKI in EHS patients when complicated with RM.

## Data Availability

The data used and/or analyzed during the current study are available from the corresponding author on reasonable request.
